# Specifics of attitudes towards traditional Chinese medicine in dental students

**DOI:** 10.1192/j.eurpsy.2022.1725

**Published:** 2022-09-01

**Authors:** E. Nikolaev, S. Petunova

**Affiliations:** Ulianov Chuvash State University, Social And Clinical Psychology Department, Cheboksary, Russian Federation

**Keywords:** Traditional Chinese medicine, dental students, attitudes

## Abstract

**Introduction:**

Traditional Chinese medicine (TCM), conceived in the womb of Chinese culture, is gaining more and more popularity in the world. What views on its possibilities do dentists studying in Russia have?

**Objectives:**

Our goal is to establish the peculiarities of the attitude to TCM that are characteristic of dental students and correlate them with their psychosocial qualities.

**Methods:**

We surveyed anonymously 106 dental students of Ulianov Chuvash State University using the Attitude to TCM Survey (E. Nikolaev) and the Sociocultural Health Questionnaire (E. Nikolaev). To analyze the interrelations, we used a correlation analysis.

**Results:**

More than two thirds of the respondents (72.6%) know about TCM, 20.8% consider it more effective than conventional medicine. Respondents with a higher level of stress show more interest in TCM (r=.27, p<.05), those who smoke hookah have less interest (r=-.25, p<.05). Students who less often work out in a gym are ready to turn to TCM (r=.23, p<.05). Students who are less often engaged in sports are more inclined to go to China for TCM treatment (r=.19, p<.05). They also less often agree that TCM can help Russian people (r=-.22, p<.05). Stronger belief in the possibilities of TCM correlates with deeper trust in private medicine (r=.22, p<.05). We did not find any correlations with the level of health.

**Conclusions:**

A more positive attitude to TCM in dental students is correlated with less physical activity, higher stress, as well as deeper trust in private medicine.

**Disclosure:**

No significant relationships.

